# Detection of inflammasome activation in liver tissue during the donation process as potential biomarker for liver transplantation

**DOI:** 10.1038/s41420-024-02042-y

**Published:** 2024-05-30

**Authors:** Sandra V. Mateo, Daniel Vidal-Correoso, Ana M. Muñoz-Morales, Marta Jover-Aguilar, Felipe Alconchel, Jesús de la Peña, Laura Martínez-Alarcón, Víctor López-López, Antonio Ríos-Zambudio, Pedro Cascales, José A. Pons, Pablo Ramírez, Pablo Pelegrín, Alberto Baroja-Mazo

**Affiliations:** 1 Molecular Inflammation Group, University Clinical Hospital Virgen de la Arrixaca, Biomedical Research Institute of Murcia (IMIB-Pascual Parrilla), 30120 Murcia, Spain; 2 General Surgery and Abdominal Solid Organ Transplantation Unit, University Clinical Hospital Virgen de la Arrixaca, 30120 Murcia, Spain; 3 Patology Unit, University Clinical Hospital Virgen de la Arrixaca, 30120 Murcia, Spain; 4 Hepatology and Liver Transplant Unit, University Clinical Hospital Virgen de la Arrixaca, 30120 Murcia, Spain; 5 Department of Biochemistry and Molecular Biology B and Immunology, Faculty of Medicine, University of Murcia, 30120 Murcia, Spain

**Keywords:** Predictive markers, Liver diseases

## Abstract

Deceased donor liver transplantation (LT) is a crucial lifesaving option for patients with end-stage liver diseases. Although donation after brain death (DBD) remains the main source of donated organs, exploration of donation after circulatory death (DCD) addresses donor scarcity but introduces challenges due to warm ischemia. While technical advances have improved outcomes, challenges persist, with a 13% mortality rate within the first year. Delving into liver transplantation complexities reveals the profound impact of molecular signaling on organ fate. NOD-like receptor family pyrin domain containing 3 (NLRP3) inflammasome activation play a pivotal role, influencing inflammatory responses. The NLRP3 inflammasome, found in hepatocytes, contributes to inflammation, fibrosis, and liver cell death. This study explores these dynamics, shedding light on potential biomarkers and therapeutic targets. Samples from 36 liver transplant patients were analyzed for ASC specks detection and inflammasome-related gene expression. Liver biopsies, obtained before and after cold ischemia storage, were processed for immunofluorescence, qRT-PCR, and Western blot. One year post-LT clinical follow-up included diagnostic procedures for complications, and global survival was assessed. Immunofluorescence detected activated inflammasome complexes in fixed liver tissues. ASC specks were identified in hepatocytes, showing a trend toward more specks in DCD livers. Likewise, inflammasome-related gene expression analysis indicated higher expression in DCD livers, decreasing after cold ischemia. Similar results were found at protein level. Patients with increased ASC specks staining exhibited lower overall survival rates, correlating with *IL1B* expression after cold ischemia. Although preliminary, these findings offer novel insights into utilizing direct detection of inflammasome activation in liver tissue as a biomarker. They suggest its potential impact on post-transplant outcomes, potentially paving the way for improved diagnostic approaches and personalized treatment strategies in LT.

## Introduction

Deceased donor liver transplantation (LT) stands as a lifesaving therapeutic option for patients with end-stage liver diseases [1]. This intricate surgical procedure has revolutionized the field of medicine, providing hope to countless individuals suffering from a spectrum of liver pathologies, including cirrhosis, hepatocellular carcinoma, and acute liver failure [2]. However, the success of liver transplantation hinges not only on the surgical expertise of transplant teams but also on the availability of suitable donor organs [3]. The quest for an expanded pool of donor organs led to the exploration of donation after circulatory death (DCD) donors, an alternative to the traditional donation after brain death (DBD) protocol [4]. DCD transplantation offers the potential to increase the donor pool, but it comes with its own set of challenges, due to the warm ischemia time (WIT), which affects the quality of the organs before transplantation [5]. Similarly, although advances in immunosuppressive therapy have greatly influenced the development and efficacy of LT, there remains a 13% mortality rate and a 20% graft loss rate within the initial year following the transplantation. Among the most serious events are acute rejection (AR), hepatic arterial thrombosis (HAT), and complications in the biliary tract, with incidences ranging from 2.5 to 35% [6–8].

As we delve deeper into the complexities of liver transplantation, it becomes evident that the fate of the transplanted organ is profoundly influenced by the intricate interplay of molecular signals within the liver microenvironment. Emerging evidence points toward the critical role of danger-associated molecular patterns (DAMPs) in orchestrating inflammatory responses during liver transplantation [9, 10]. These endogenous molecules, released in response to tissue injury, can activate inflammasomes, particularly the NOD-like receptor family pyrin domain containing 3 (NLRP3) inflammasome [11]. The NLRP3 inflammasome constitutes a multiprotein complex within the cytoplasm that plays a crucial role in starting the inflammatory response following a stimulus [12]. The initial phase of canonical inflammasome activation commences with the triggering of Toll-like receptors (TLRs) by pathogen-associated molecular patterns (PAMPs) or DAMPs. This, in turn, results in the subsequent activation of nuclear factor kappa B (NF-kB). This priming stage leads to increased transcription of *NLRP3*, interleukin (*IL)1B*, and *IL18* [13]. Following this, DAMPs activate the sensor NLRP3 inflammasome oligomer, and the adaptor protein apoptosis-associated speck-like protein (ASC) is recruited via a pyrin domain. ASC, in turn, recruits pro-caspase 1 through Caspase Recruitment Domain (CARD)–CARD interactions. The assembly of the inflammasome results in caspase 1 activation via auto-proteolysis, ultimately triggering the generation of mature IL-1β and IL-18. This culminates in cell death through pyroptosis and the subsequent release of these cytokines. They then exert their effects on the tissue through IL-1 and IL-18 receptors, creating an inflammatory milieu through autocrine and paracrine pathways [13]. In the context of liver transplantation, the activation of NLRP3 inflammasome is of particular interest [14]. Inflammasome is mainly found in macrophages, but has also been found in other cells, such as hepatocytes, and its activation has been related to inflammation, fibrosis, and cell death in the liver [15, 16].

In this study, we utilized immunofluorescence to detect the presence of activated inflammasomes within hepatocytes by visualizing ASC “specks”, components forming part of the multiprotein complex assembled after activation. This analysis was performed directly on fixed liver tissue obtained from donated organs during the donation process. Simultaneously, we analyzed the expression of inflammasome-related genes in the same tissue, assessing the impact of donation type and cold ischemic time on both biomarkers. Importantly, the presence of ASC specks and *IL1B* gene expression were found to be associated with the short-term outcome in LT. These findings highlight the connection between liver inflammasome activation during the donation process and the dynamics of LT, offering valuable insight into their potential as biomarker and therapeutic targets.

## Results

### The type of donation does not present apparent histological differences

**Fig. 1:**
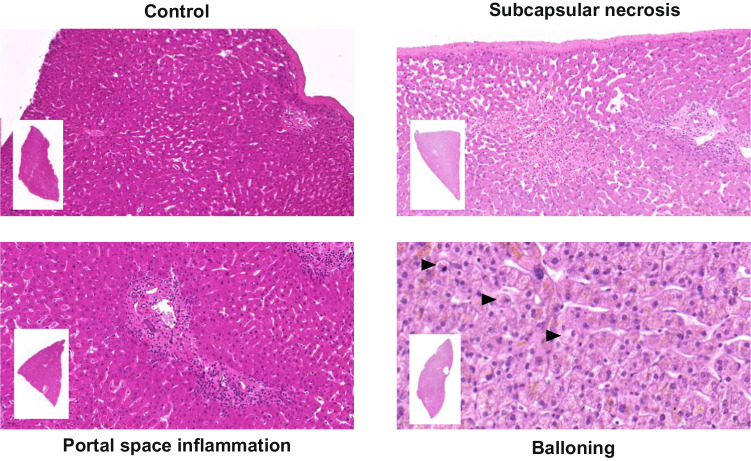
Histological evaluation of donated livers. Representative hematoxylin-eosin images of donated livers biopsies collected during liver procurement depicting subcapsular necrosis, portal space inflammation, and hepatocyte ballooning (black arrows). A control liver with a score of 0 for all analyzed histological parameters is also included.

DBD and DCD organs originate from different sources, theoretically posing worse conditions for DCD livers. However, upon histological analysis, we did not find significant differences between the two types of donations for several studied parameters (Table 1). Only subcapsular necrosis (*p* = 0.058), portal space inflammation (*p* = 0.079), and ballooning (*p* = 0.060) approached statistical significance, but notably, only subcapsular necrosis exhibited worse results in DCD organs compared to DBD (15.4% vs 0% positive determination). Surprisingly, the other two parameters appeared to be better in the DCD group (38.5% vs 50% and 7.7% vs 27.3%, for portal space inflammation and cytoplasmic ballooning, respectively) (Table 1). However, only DCD organs exhibited moderate scores in both determinations (Table 1). Representative hematoxylin-eosin pictures are presented in Fig. 1.

**Table 1:** Histological score for donated livers at recovery time.

	Score*	DBD (*n* = 22) Number (%)	DCD (*n* = 13) Number (%)	*p*
Necrosis	0	22 (100)	11 (84.6)	0.058
	1	0 (0)	2 (15.4)	
Hepatocellular acidophilic necrosis	0	22 (100)	12 (92.3)	0.187
	1	0 (0)	1 (7.7)	
Centrolobulillar necrosis	0	22 (100)	12 (92.3)	0.187
	1	0 (0)	1 (7.7)	
Subcapsular necrosis	0	22 (100)	11 (84.6)	0.058
	1	0 (0)	2 (15.4)	
Sinusoidal dilatation	0	18 (81.8)	10 (76.9)	0.594
	1	3 (13.6)	3 (23.1)	
	2	1 (4.6)	0 (0)	
Portal space inflammation	0	11 (50)	8 (61.5)	0.079
	1	11 (50)	3 (23.1)	
	2	0 (0)	2 (15.4)	
Lobular inflammation	0	19 (86.4)	11 (84.6)	0.886
	1	3 (13.6)	2 (15.4)	
Interphase activity	0	22 (100)	13 (100)	1
Macro steatosis	No	17 (77.3)	11 (84.6)	0.600
	Yes	5 (22.7)	2 (15.4)	
Cytoplasmic ballooning	0	16 (72.7)	12 (92.3)	0.060
	1	6 (27.3)	0 (0)	
	2	0 (0)	1 (7.7)	
Nuclear glycogenation	0	22 (100)	13 (100)	1
Cholestasis	0	22 (100)	13 (100)	1
Hemosiderosis	0	21 (95.4)	13 (100)	0.435
	1	0 (0)	0 (0)	
	2	1 (4.6)	0 (0)	
Portal space fibrosis	0	22 (100)	12 (92.3)	0.187
	1	0 (0)	0 (0)	
	2	0 (0)	1 (7.7)	
Lobular fibrosis	0	22 (100)	13 (100)	1
Ceroid pigment	0	21 (95.4)	13 (100)	0.435
	1	1 (4.6)	0 (0)	

*0 = no presence; 1 = mild; 2 = moderate.

### The activated inflammasome multi-protein complex can be detected in fixated liver biopsies by immunofluorescence

**Fig. 2:**
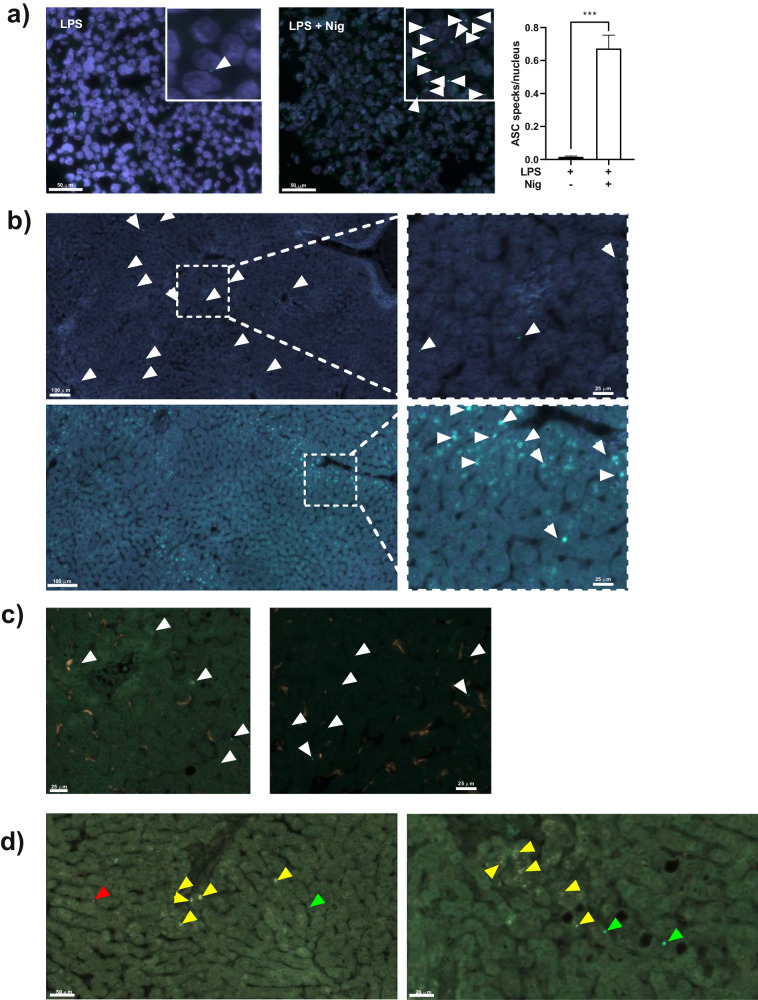
Detection of ASC specks in fixed tissue from donated livers. Representative images of ASC specks, indicating the presence of activated inflammasomes, detected by immunofluorescence in THP-1 cells (**a**) or liver tissue (**b**–**d**). **a** THP-1 cells were in vitro primed with LPS for 4 hours and then incubated in the presence or absence of nigericin for an additional 1 hour. Following stimulation, the cells were fixed, centrifuged, and embedded in a specialized processing gel. The gel block was then processed, including fixation in PaxGene medium and paraffin embedding. The white-lined square in the upper right corner represents a magnification of the image. White arrowheads indicate the presence of ASC specks represented as a green dot. The bar-graph depicts the quantification of ASC specks per nucleus in three independent images. ***p ≤ 0.001. **b** Two different patterns of ASC speck staining can be found in fixed liver tissues. In the upper picture, green dots, indicated by white arrowheads, are detected, while in the lower picture, a reticular structure surrounding the nucleus is shown. A magnification of each image is shown on the right side. Two representative images are shown. **c** Liver biopsy samples were double counter-stained with anti-ASC antibody (green) and anti-CD68 antibody (red) (Invitrogen; #14-0688-82). White arrowheads indicate the presence of ASC specks. **d** Liver biopsy samples were double counter-stained with anti-ASC (green) and anti-active IL-1β (red) (Invitrogen; #PA5-105048). Green arrowheads signal single staining for ASC specks, red arrowheads indicate the presence of single staining for active IL-1β, while yellow arrowheads show co-staining for both markers. Two representative images are shown.

In previous investigations, our group identified a variety of DAMPs released into end-ischemic organ preservation solution (eiOPS) during the cold ischemic storage of donated livers [9, 10]. Subsequently, we conducted in vitro experiments demonstrating that several of these DAMPs had the capacity to activate the NLRP3 inflammasome in myeloid cells [9]. Nevertheless, the direct activation of the inflammasome within liver tissue during the donation process remained unexplored. In this context, the current study employed an antibody targeting the adaptor protein ASC, which allowed us to visualize the formation of the large multi-protein inflammasome complex, commonly referred to as “speck” [17]. As depicted in Fig. 2a, the anti-ASC clone O93E9 (Cat#676502, Biolegend) effectively stained ASC specks in THP1 cells that were incubated with LPS plus nigericin (an established control for NLRP3 inflammasome activation [18]), and treated to mimic liver biopsies (preserved using PaxGene fixation and paraffin embedding). Thus, our next aim was to detect ASC specks in liver biopsies obtained from donated organs through immunofluorescence analysis. In this case, we were able to visualize inflammasome activation by immunostaining of ASC specks in fixed liver tissue (Fig. 2b). The ASC specks staining was observed as a singular dot within the cytoplasm, situated near the cell membrane, while also forming a reticular structure surrounding the nucleus (Fig. 2b, up and down, respectively). Notably, hepatocytes were the exclusive cell type displaying staining for ASC specks, whereas other cells expressing the NLRP3 inflammasome, such as macrophages, did not exhibit this phenomenon (Fig. 2c). It is worth to note that by employing an antibody that recognizes active IL-1β, we observed some co-localization between cleaved IL-1β and ASC specks (Fig. 2d and Figure S1).

### DCD livers exhibit a tendency to have more ASC specks than DBDs

**Fig. 3:**
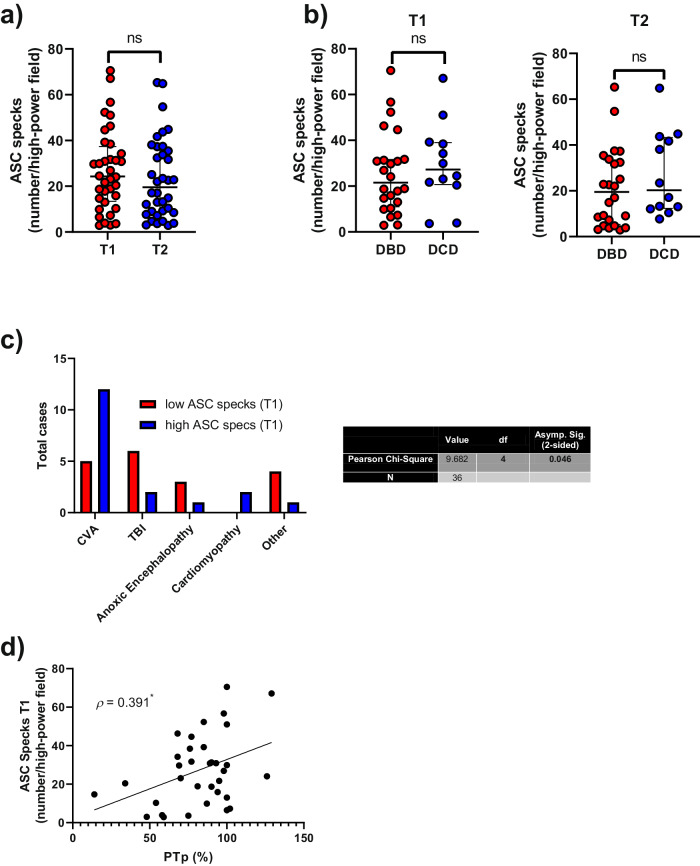
Quantification of ASC specks in liver biopsies from donated livers. **a**, **b** Mean number of ASC specks detected per high-power field in liver biopsies from the 36 donated livers, comparing those collected before liver extraction (T1) with those collected after cold ischemia storage (T2) (**a**), and between DBD and DCD group at both collection times (**b**); ns = non-significant. Each point represents a unique subject/patient. Results are presented as median with interquartile range. **c** Total number of donors with a high (> median) or low (< median) number of ASC specks detected per high-power field in liver biopsies collected before liver recovery (T1), categorized by the cause of death. **d** Pearson’s correlation illustrating the relationship between the mean number of ASC specks detected per high-power field in liver biopsies and the percentage of prothrombin time; *p ≤ 0.05.

ASC specks were identified in liver tissue collected at two different time points: before (T1) and after (T2) cold ischemic storage. Our analysis did not reveal any significant differences in the number of ASC specks detected in tissue liver between these two-time points (Fig. 3a) or even between DBD and DCD livers (Fig. 3b). However, it is worth noting that there was a slight tendency toward fewer specks in T2, while more specks were counted in DCD livers. Furthermore, the cause of death showed a significant correlation with the number of ASC specks detected before cold ischemic storage (*p* = 0.046), with higher accumulations after acute cerebrovascular accident (CVA) and cardiomyopathies (Fig. 3c). Similar results were observed for the percentage of donor prothrombin (PTp) activity (Fig. 3d). However, no other liver function tests (LFTs) presented significant correlation with ASC specks.

### eiOPS induces NLRP3 inflammasome oligomerization in transfected hepatocyte-derived Huh7 cell line

**Fig. 4:**
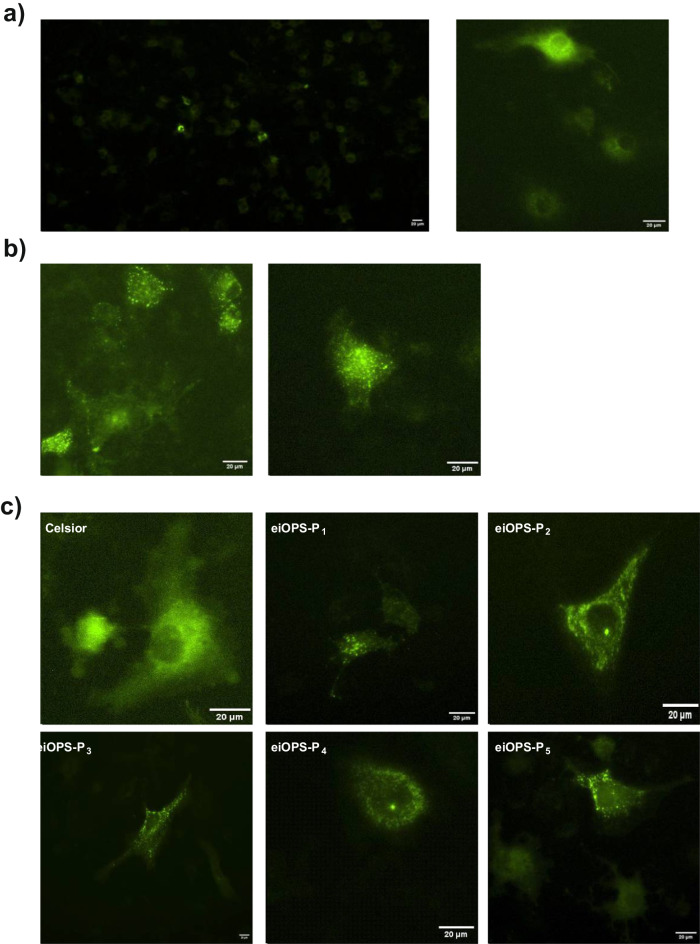
Organ preservation solution collected after cold ischemic storage activates the NLRP3 inflammasome in hepatocytes. Representative images of the human hepatocyte-like Huh7 cell line transfected with YFP-tagged NLRP3 protein in resting conditions (**a**), stimulated with nigericin (**b**) or incubated with eiOPS from different donated livers (P_1-5_) or Celsior as negative control (**c**). Representative pictures were taken after 2 hours (**a, b**) or 4 hours (c) of incubation, respectively.

Given these results, we wondered whether DAMPs presents in eiOPS could directly activate the inflammasome in hepatocytes. As displayed in Fig. 4a, human hepatocyte-derived Huh7 cells transfected with recombinant NLRP3 inflammasome fused to YFP exhibited green fluorescence throughout the cytosol. Upon incubation with the potassium ionophore nigericin, a potent NLRP3 activator, activated cells displayed a punctuated staining pattern, indicating NLRP3 inflammasome oligomerization (Fig. 4b). Furthermore, a similar activated pattern appeared when cells were incubated with eiOPS collected from different donated livers, as compared to Celsior solution used as a negative control (Fig. 4c).

### Expression of NLRP3 inflammasome-related genes is higher in DCD livers but decreases after cold ischemia storage

**Fig. 5:**
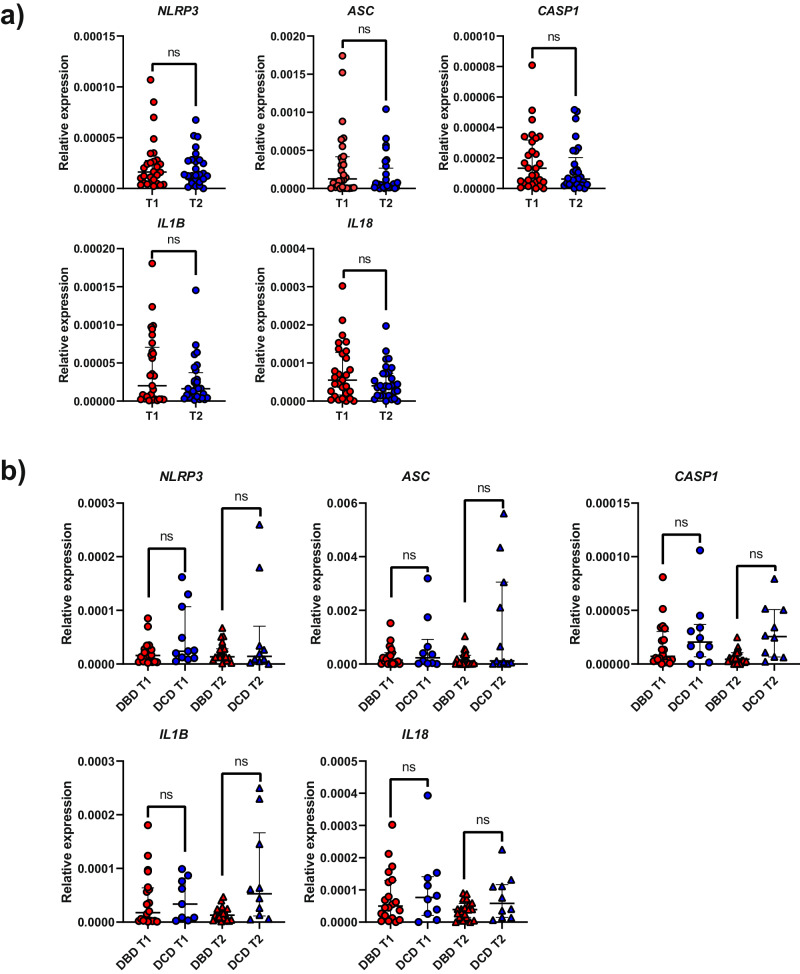
Expression of NLRP3 inflammasome-related genes in liver biopsies from donated livers. Relative expression of *NLRP3*, *ASC*, *CASP1*, *IL1B* and *IL18* in liver biopsies from the 36 donated livers, comparing those collected before liver extraction (T1) with those collected after cold ischemia storage (T2) (**a**), and between DBD and DCD group at both collection times (**b**). Each point represents a unique subject/patient. Results are presented as a median with a interquartile range.

Furthermore, we aimed to investigate the expression of genes associated with the NLRP3 inflammasome, including *NLRP3* as the sensor, *ASC* as the adaptor protein, *CASP1* as the executioner, and pro-inflammatory cytokines *IL1B* and *IL18*, within liver tissue. Our primary focus was to determine whether the expression levels of these genes underwent significant changes during the organ donation process. Our analysis revealed that parallel to the results observed for ASC speck detection, the gene expression of all these proteins showed a similar pattern in tissues collected both before and after cold ischemia. Nevertheless, there was a perceptible trend towards lower expression in T2 samples (Fig. 5a). When we analyzed the influence of the type of donation, we did not identify significant differences in gene expression between T1 and T2 biopsies (Fig. 5b). However, again, DCD samples exhibited a slightly higher gene expression of all inflammasome-related proteins, observed in both for T1 and T2 samples (Fig. 5b).

**Fig. 6:**
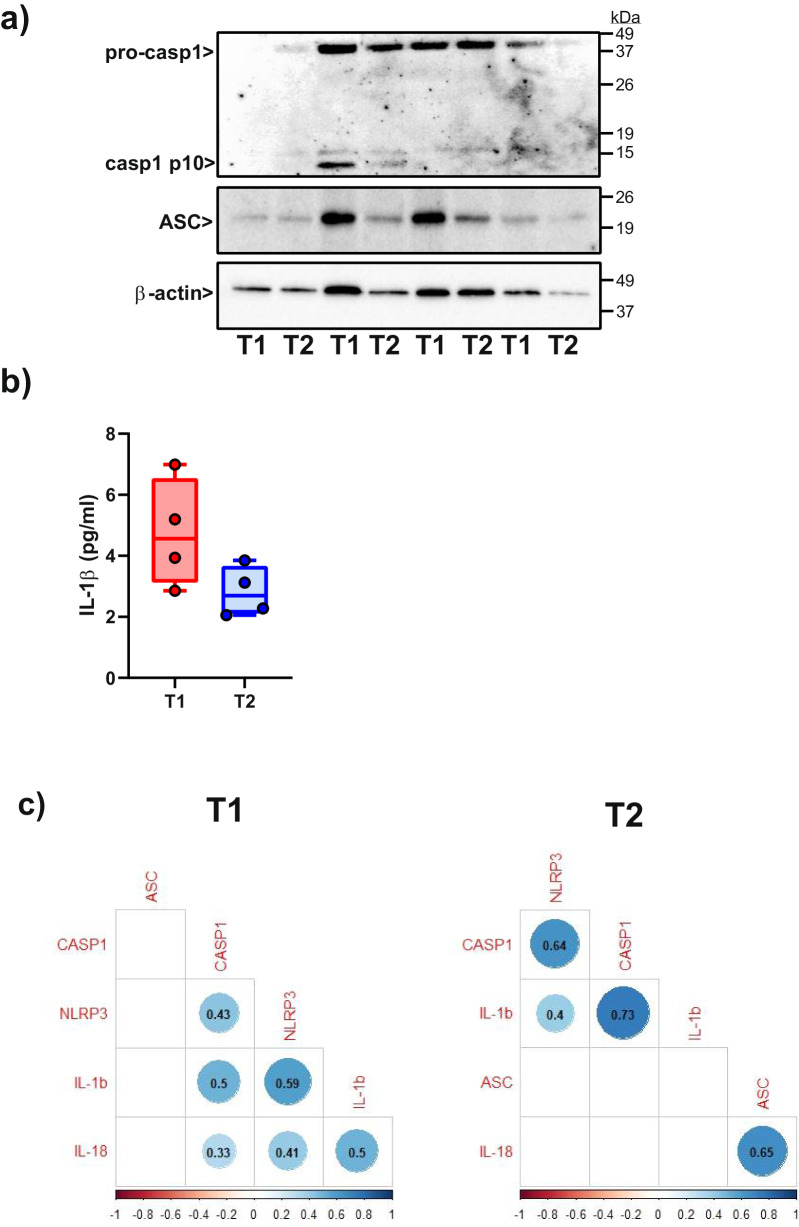
Detection of ASC, caspase-1 and IL-1β in liver biopsies at the protein level. **a** Representative western blot for the detection of caspase-1 (pro- and active forms) and ASC in liver biopsy extracts from 4 different donated livers collected before donation (T1) or after cold ischemic storage (T2). Β-actin was used as a loading control. Full-length western blots are published as a Supplementary File. **b** Concentration of IL-1β in the 4 extracted tissues as detected by ELISA. **c** Correlation matrix among the expression of different NLRP3 inflammasome-related genes detected in liver tissue collected before donation (T1) or after cold ischemic storage (T2).

Given the relatively low gene expression levels of most of these proteins, we questioned whether this was sufficient to detect the proteins within liver tissue. Notably, we successfully detected the expression of ASC and Caspase-1 through Western blotting (Fig. 6a), while IL-1β was detected via ELISA (Fig. 6b). Additionally, in some samples, we observed the presence of the active p10 form of Caspase-1 (Fig. 6a). Similar to gene expression, there was a trend toward lower protein expression in T2 samples (Figure S2 and Fig. 6b).

Unfortunately, we did not observe a significant correlation between cold ischemic time (CIT) and gene expression in T2 samples, nor did we find any correlation between the number of ASC specks and the gene expression of the individual proteins studied. Likewise, no correlation was identified between RNA expression in T1 samples and WIT, the cause of death or pre-donation LFTs. Nevertheless, we discovered a robust correlation among the expression levels of all the inflammasome-related proteins, particularly at each specific time point (Fig. 6c).

### Poorer global survival of LT patients is consistent with a higher detection of ASC specks in hepatocytes of the donated organs

**Fig. 7:**
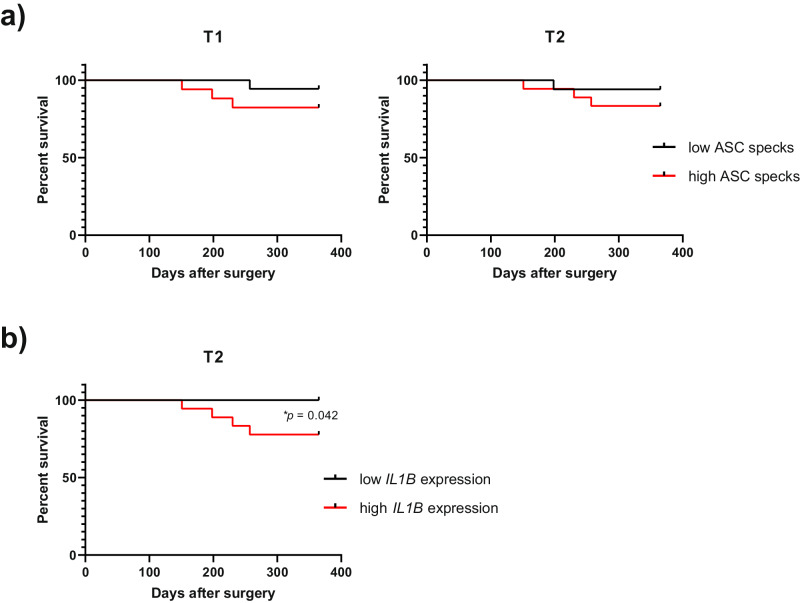
Hepatocyte inflammasome activation detection during donation as biomarker for liver transplant outcome. Kaplan-Meier survival curve for the presence of high (> median) or low (< median) amounts of ASC specks (**a**) or *IL1B* gene expression (**b**) in donated fixed liver tissue collected before donation (T1) or after cold ischemic storage (T2).

In order to investigate the potential influence of tissue biopsy ASC specks and inflammasome-related gene expression on liver transplant outcomes, we conducted a one-year follow-up of 36 patients whose eiOPS was analyzed. The post-transplant evolution revealed various complications with incidence in the morbi/mortality of patients. Thus, approximately 17% of patients experienced AR, 6% suffered from HAT, 19% encountered biliary complications, primarily anastomotic strictures (8.3%), 5.6% had severe post-reperfusion syndrome, and none experimented primary graft dysfunction. Furthermore, 3 patients required re-transplantation due to graft loss, and 2 patients died within this timeframe (Table 2). In this regard, the presence of ASC specks detected in liver biopsies both before and after cold ischemic storage, was found to be associated with global survival during the first year post-transplantation (Fig. 7a). Although not statistically significant, a higher presence of ASC specks correlated with poorer survival. Furthermore, the expression of *IL1B* in liver biopsy after cold ischemic storage significantly associated with global survival, with all events (organ loss or death of patient) corresponding to high *IL1B* expression (Fig. 7b). Additionally, early allograft dysfunction, a critical factor influencing graft and patient outcomes, was assessed using the MEAF score [19] (Table 2). However, we did not find a correlation with any of the measured variables.

**Table 2:** One-year follow-up of the 36 liver transplant patients included in the study.

Post-transplant event	N (%)
Acute rejection	6 (16.7)
Hepatic artery thrombosis	2 (5.6)
Biliary complications	
Anastomotic strictures	3 (8.3)
Leaks	2 (5.6)
Cholangitis	1 (2.8)
Cholestasis	1 (2.8)
Severe post-reperfusion syndrome*	2 (5.6)
Primary graft dysfunction	0
Graft loss (re-transplantation)	3 (8.3)
Deceased	2 (5.6)
	**Median (range)**
MEAF score	2.26 (3.92-4.29)

*Severe events were defined by greater hemodynamic instability, a drop in MAP/HR exceeding 30% of baseline, asystole or hemodynamically significant arrhythmias; or the need to start the infusion of vasopressors during the intraoperative period and to continue throughout the postoperative period (Hilmi et al. 2008. Liver Transplant; DOI: 10.1002/lt.21381).

## Discussion

The reintroduction of DCD organs has resulted in increased organ donations in several countries [4], with notable success in Spain [20]. Traditionally, DCD has been associated with longer WIT and a higher incidence of complications compared to DBD grafts [5]. However, advances in procurement and preservation techniques have facilitated improved outcomes and broader utilization of DCD donors [21]. The histological analysis illustrates similarities between both types of donations. Furthermore, DBD livers exhibited worse scores compared to DCD, a trend noted previously, particularly when DCD is combined with normothermic regional perfusion (NRP) [22]. This observation is particularly noteworthy, given the exceptionally brief WIT in the current cohort.

Our focus has been on identifying biomarkers to deepen our understanding of the distinct mechanisms inherent in the donation process, ultimately aiming to enhance the quality of donated organs. Previous findings detailed how CIT and DCD adversely impacted the release of various DAMPs into the OPS, with potential implications for early graft function [9, 10]. Furthermore, we demonstrated that specific DAMPs released into the OPS could activate the NLRP3 inflammasome in macrophages [9]. While many experiments involved in vitro activation of human monocytes in the presence of eiOPS, preliminary data also indicated a possible direct inflammasome activation in liver tissue [9]. Detection of ASC specks by immunofluorescence is a hallmark of inflammasome activation [17], and in situ detection in tissue may be important for the study of physiological inflammatory processes, including liver transplantation [14]. Using a specific antibody clone against ASC protein (O93E9), we detected ASC specks in hepatocytes from fixed liver biopsies obtained during the donation process. The specificity of active inflammasome staining was demonstrated when active IL-1β staining merged with ASC specks. After activation, NLRP3 Pyrin Domain (PYD) filament recruits ASC by nucleating the ASC PYD filament. The CARD of ASC also clusters and forms a filament. The ASC CARD filament recruits caspase-1 by nucleating the caspase-1 CARD filament. The caspase-1 caspase domain (p20/p10) dimerizes and autoprocesses, resulting in its activation. The active caspase-1 then cleaves pro-cytokines in the IL-1 family to generate mature cytokines [23, 24]. Moreover, this co-localization appears either when ASC specks are detected as a singular dot within the cytoplasm or when forming a reticular structure surrounding the nucleus. This last type of structure has been showed before in cardiac [25] and lung tissue [26]. In the heart, apparently well-defined ASC specks were shown, but using a polyclonal rabbit antibody with no catalog reference published, while not as well-defined pictures were presented in lung tissue from patients with chronic obstructive pulmonary disease using another rabbit polyclonal antibody from Adipogen (Cat# AG-25B-0006).

Controversially, our liver samples exhibited an absence of macrophage staining indicative of inflammasome activation. This finding contrasts with our previous publication [9], where staining using a polyclonal anti-ASC antibody from SantaCruz Biotechnologies (Cat# Sc-22514-R; RRID: AB_2174874) revealed widespread cytosolic immunohistochemical staining in Kupffer cells, with no detection of ASC specks. Similar outcomes were observed by Hong et al. in a rat LT model [27]. However, in both cases, no specks were identified as those observed when utilizing clone O93E9, which seems to exclusively recognize ASC oligomers rather than cytosolic monomers. The absence of ASC specks in Kupffer cells may be attributed to the rapid induction of pyroptosis in myeloid cells following inflammasome activation [28]. Pyroptotic cell death is orchestrated by the gasdermin (GSDM) D protein, initially processed by inflammasome-activated caspase-1 [29]. Caspase-1, beyond pro-IL-1 family cytokines, also cleaves GSDMD, releasing its necrotic and cytotoxic N-terminal domain from its repressor C-terminal domain. Subsequently, the N-terminal domain of GSDMD binds to the plasma membrane, forming pores through homo-oligomerization [30]. The permeabilization of the plasma membrane by GSDMD pores allows water entry into the cytosol, triggering cellular blebbing and plasma membrane rupture, ultimately leading to pyroptosis, further mediated by the oligomerization of nerve injury-induced protein 1 [31]. Finally, pyroptotic cells undergo bursting, resulting in the release of intracellular components, including the NLRP3 oligomeric inflammasome itself, creating a highly pro-inflammatory environment [32]. While this phenomenon has also been observed in hepatocytes [16], the cell death in hepatocytes is not as rapid as in macrophages [33]. However, hepatocytes pyroptosis could be associated with the lower presence of ASC specks and inflammasome-related gene expression in liver tissue after cold ischemia storage. Despite our anticipation that the cold ischemia process and the release of DAMPs into the eiOPS during this period would impact NLRP3 inflammasome activation, the observed effects were not as expected. Liver samples collected just before procurement from deceased donors exhibited significant levels of activated inflammasome and inflammation. Moreover, the cause of death and pre-donation prothrombin time of donors exhibited a positive association with higher amounts of ASC specks in these liver samples. Ischemic stroke [34, 35] and cardiomyopathies [36] have been clearly linked to NLRP3 inflammasome activation. Serum NLRP3 has been identified in patients with acute ischemic stroke [37], as well as in individuals with different cardiomyopathies [38, 39], and has been associated to disease progression. Extracellular NLRP3 oligomeric particles can be internalized by other cells, activating their inflammasome machinery [16, 32]. Nevertheless, DAMPs have also been identified in the plasma of deceased donors [40]. Additionally, thrombin present the capability to activate the NLRP3 inflammasome through engagement of G protein-coupled receptors and the induction of intracellular reactive oxygen species [41].

On the other hand, ASC specks and gene expression was relatively higher in DCD organs, possibly linked to the elevated concentration of various DAMPs capable of activating the inflammasome in this type of donation [9, 10]. Furthermore, hepatocyte NLRP3 inflammasome activation could potentially be directly triggered by DAMPs present in the eiOPS. Although the presented model using transfected cells may not be entirely physiological, it has been utilized extensively in previous studies [42, 43], including those involving cultured hepatocytes [16].

This inflammasome activation occurred during donation and cold preservation directly into the tissue may influence the short-term outcome of the LT. Thus, the presence of ASC specks detected by immunofluorescence or the expression of *IL1B* gene could be used as potential biomarkers for the evolution of the patients during the first year post-transplantation. The NLRP3 inflammasome plays a role in ischemia-reperfusion injury [44] and early inflammation and rejection after LT [27]. Therefore, beyond its utility as a biomarker, inhibiting the inflammasome could be a crucial strategy to mitigate the damage caused by deceased donation and organ storage, potentially enhancing both the quality of marginal livers and the outcomes of liver grafts. The synergy of employing NRP to prevent warm ischemia and ex vivo machine perfusion to counter static storage presents an optimal scenario for the future of liver transplantation in effectively managing inflammasome activation [14]. Extracorporeal normothermic machine perfusion, which sustains organs at body temperature while supplying oxygen and nutrients until implantation [45], could serve as a versatile platform for testing therapeutic drugs during ex vivo liver preservation [46]. Additionally, NRP could act as an initial delivery pathway for drugs specifically designed to inhibit the inflammasome pathway in the donor [14].

Nevertheless, the current study has certain limitations. Although important, the utilization of immunohistochemistry as a biomarker tool necessitates thorough standardization and validation processes [47, 48]. Nonetheless, the integration of other technologies, such as tissue RNA expression analysis, can complement and enhance this capability [49, 50]. Likewise, including super rapid recovery (SRR) patients in the analysis may introduce additional confounding. NRP have been demonstrated to reduce biliary complications and improve DCD liver graft survival [51, 52]. However, in our cohort, we observed no noticeable differences between NRP and SRR from DCD livers due to the limited number of SRR donors (data not shown). Nevertheless, CIT remains an independent predictive factor of graft loss among DCD livers even when NRP is employed [53]. Additionally, the sample size was notably limited, and although our cohort reasonably aligns with the current LT follow-up at our hospital [54], it is crucial to validate these findings in new patient cohorts before considering their potential integration into clinical practice.

Overall, these findings underscore the molecular connection between NLRP3 inflammasome activation during donation and LT outcomes. Various factors such as the type of donation, cause of death, and liver function markers from donors can influence inflammasome activation within the donated liver, potentially affecting post-transplant progression. Furthermore, these data hold promise for novel therapeutic interventions aimed at inhibiting inflammasome activation throughout the donation process, with the aim of enhancing current LT outcomes.

## Patients and Methods

### Patients

Samples from 36 patients who underwent transplantation between July 2020 and July 2022 were included. Only patients whose samples could be processed and stored within the first 12 hours by the IMIB-Biobank were included. Table 3 provides a summary of the demographic and clinical characteristics of both donors and recipients. In brief, 23 explanted livers came from DBD donors (63.9%). In DCD donors (*n* = 13; 36.1%), 86.4% were recovered under NRP, whereas only 2 livers were recovered with the SRR technique. The principal cause of death was CVA (*n* = 17; 47.2%), followed by traumatic brain injury (TBI) (*n* = 8; 22.2%). The mean age of donors was 55.95 ± 14.82 years old, and male represented 61.1%. CIT reached a mean of 269.50 ± 143.14 min. No significant differences were found between the different types of donations in terms of age (*p* = 0.611), sex (*p* = 0.134), body mass index (*p* = 0.203), CIT (*p* = 0.349), cause of death (*p* = 0.117) of pre-donation LFTs (*p* ≥ 0.584). Recipients had a mean age of 60.97 ± 6.13 years old, with 77.8% being males. The main etiology for transplantation was alcoholic cirrhosis (55.6%), followed by viral infection (11.1%). Nevertheless, 4 patients (11.1%) were treated with a re-transplant, and 1 (2.8%) died at surgery.

**Table 3:** Demographic characteristics of 36 organ donors and recipients included in the study.

Variables	Donors (*n* = 36)		*P*	Recipients (*n* = 36)
	DBD (*n* = 23)	DCD (*n* = 13)		
Age	55.30 ± 14.65; 54 (25-77)	57.85 ± 13.58; 62 (26-76)	0.611^a^	60.97 ± 6.13; 61 (48-73)
Sex				
Male	12 (52.2)	10 (76.9)	0.134^b^	28 (77.8)
Female	11 (47.8)	3 (23.1)		8 (22.2)
BMI	27.02 ± 3.98; 26.65 (19.23-37.04)	25.15 ± 4.35; 24.69 (18.41-34.60)	0.203^a^	26.87 ± 4.77; 26.77 (19.82-37.34)
CIT (min)	284.90 ± 174.80; 245 (30-560)	244.60 ± 66.06; 240 (180-395)	0.349^a^	
Cause of death				
CVA	12 (52.2)	5 (38.5)	0.117^b^	
TBI	7 (30.4)	1 (7.7)		
Anoxic encephalopathy	2 (8.7)	2 (15.4)		
Cardiomyopathy	0	2 (15.4)		
Other	2 (8.7)	3 (23.1)		
Pre-donation LFTs				
AST (U/L)	79.1 ± 116; 32 (11-442)	59.5 ± 69.4; 28 (10-260)	0.584^a^	
ALT (U/L)	53.7 ± 76.4; 21.5 (8-270)	57.8 ± 64.5; 37 (8-202)	0.874^a^	
PTp activity (%)	82.8 ± 23.7; 89.5 (14-126)	80.6 ± 24; 76.5 (34-129)	0.795^a^	
NRP		11 (84.6)		
Functional WIT (min)		13.50 ± 6.49; 14 (2-25)		
Diseases				
Alcoholic cirrhosis				20 (55.6)
HCV				4 (11.1)
Ischemic cholangiopathy				3 (8.3)
Cryptogenic liver Cirrhosis				2 (5.6)
NASH				2 (5.6)
Other				5 (13.8)
Re-transplant patients				4 (11.1)
Intraoperative death				1 (2.8)

Continuous variables are expressed as mean ± SD; median (range). Qualitative variables are expressed as frequency (%). *DBD* donation after brain death, *DCD* donation after circulatory death, *BMI* body mass index, *CIT* cold ischemia time, *CVA* acute cerebrovascular accident, *TBI* traumatic brain injury, *LFTs* liver function tests, *AST* aspartate aminotransferase, *ALT* alanine aminotransferase, *PTp* percentage of prothrombin time, *NRP* normothermic regional perfusion, *WIT* warm ischemia time, *HCV* hepatitis C virus, *HBV* hepatitis B virus, *NASH* non-alcoholic steohepatitis. ^a^T-test. ^b^Chi-square test.

### Liver biopsies procurement

A small piece of biopsy from a donated organ was taken at 2 different times. First, a biopsy was taken before liver procurement (T1), whereas a second biopsy was taken after static cold ischemic storage, just before implantation in the recipient (T2). Both pieces were fixed in PaxGene Tissue Containers (PreAnalytiX GmbH, Feldbachstrasse, Switzerland) and embedded in paraffin.

### Clinical follow-up of transplant patients

The clinical follow-up protocol for patients undergoing liver transplantation in our unit include: Daily analytical monitoring during their stay in the ICU and every 48 hours during their hospital stay. Weekly abdominal Doppler ultrasonography measuring flows and resistive index of the portal vein, hepatic artery, and hepatic veins. For the diagnosis of hepatic arterial thrombosis, in cases of clinical suspicion due to elevated liver enzymes, fever with bacteremia, cholangitis, sepsis, or other clinical indications, we repeat Doppler ultrasonography of the hepatic artery, computed tomography (CT) with the arterial angiographic study of the celiac trunk, and superior mesenteric artery. In cases of clinical suspicion of biliary stenosis, an ultrasound study of the hepatic graft and magnetic resonance cholangiopancreatography are performed. In the case of non-anastomotic stenosis, a CT with angiographic study of the celiac trunk and superior mesenteric artery is carried out. For the diagnosis of acute rejection, a liver biopsy is performed with ultrasound control, and the Banff criteria are followed for classification [55]. Global survival during the first year post-transplantation was defined as either graft loss necessitating re-transplantation or patient death.

### ASC specks detection

For immunofluorescence images of human macrophage THP-1 cultured cells (American Type Culture Collection Cat# TIB­202), the following procedure was followed: THP-1 cells were initially seeded in 6-well culture plates with complete medium (RPMI supplemented with 2 mM of L-glutamine and 10% fetal bovine serum (FBS)) and incubated at 37 °C for 30 min with 0.5 µM phorbol 12-myristate 13-acetate (PMA). Then, the cells were primed with 500 ng/mL of LPS for 4 hours. After the priming step, the supernatants were carefully aspirated, and the cells were washed twice with physiological ET buffer [56], followed by incubation in the presence or absence of 10 µM nigericin for 1 h at 37 °C. Following stimulation, the cells were fixed for 15 min at room temperature using 4% formaldehyde in phosphate-buffered saline (PBS). The fixed cells were then centrifuged to form a pellet, which was subsequently included in a specialized processing gel (Histogel, Cat# HG-4000-012, ThermoFisher Scientific, Walthan, USA), according with the manufacturer´s protocol. The gel block was then processed as a typical tissue sample, including fixation in PaxGene medium and paraffin embedding.

After deparaffination and rehydration, 3-μm-thick liver biopsy or tissue-like processed THP-1 cells sections underwent a heat-induced antigen retrieval procedure (Dako PT-Link, Agilent, Santa Clara, USA) while maintaining a basic pH throughout. The samples were subsequently blocked for 1 hour using 30 µl of Normal Goat Serum Blocking Solution, 2.5% (Vector Laboratories, Cat# S-1012-50) in a humidity cabinet at 37 °C. Following this, they were incubated overnight with the primary mouse monoclonal anti-ASC antibody (diluted at 1:250; Biolegend Cat# 676502, Clone O93E9), and then washed three times for 10 min each with PBS. Then, the samples were incubated for 1 hour with a fluorescence-conjugated secondary antibody (Alexa 488 Goat anti-mouse, diluted at 1:1000; Invitrogen Cat# A-11001). Coverslips were mounted using DAPI Fluoromount-G (SouthernBiotech Cat# 0100-20) before imaging. The slides were digitalized using a high-resolution slide scanner (Pannoramic MIDI II, 3D Histech, Budapest, Hungary), equipped with dedicated software (Pannoramic Scanner 3.0.2, 3D Histech). The digitalized sections were examined, and representative images were obtained by using a specialized software (Slide Viewer, Ver. 2.6.0.166179, 3D Histech). ASC quantification was represented as the mean number of ASC specks per high-power field, averaged from 10 randomly selected high-power fields per image.

On the other hand, human hepatocyte-derived cellular carcinoma Huh7 cell line (kindly donated by Dr. Águeda González from Instituto de Investigaciones Biomédicas Alberto Sols, Madrid, Spain) was maintained in Dulbecco’s modified Eagle’s medium (DMEM) Hihg glucose w/L-Glutamine w/o Sodium Pyruvate (Biowest, Nuaillé, France) supplemented with 10% FBS, 2 mM GlutaMAX (Life Technologies), and 1% penicillin-streptomycin (Life Technologies). 5 × 10^4^ Huh7 cells were seeded in 24-well culture plates. Lipofectamine 2000 was used for the transfection of Huh7 cells with a plasmid expressing yellow fluorescent protein (YFP)-tagged human NLRP3 protein [43], according to the manufacturer’s instructions. After 24 h, cells were stimulated with different treatments per duplicate. On the one hand, cells were incubated with Celsior solution (IGL, Lissieu, France) as a negative control. On the other hand, NLRP3 inflammasome oligomerization was induced with nigericin 10 µM (Sigma-Aldrich) as a positive control. Likewise, eiOPS from different donated livers were used [9]. Imaging was carried out 2 and 4 hours after the addition of the different treatments with a Nikon Eclipse Ti microscope equipped with ×20 S Plan Fluor objective (numerical aperture, 0.45) and a digital Sight DS-QiMc camera (Nikon, Tokyo, Japan) with 387/447 nm and 472/520 nm filter sets (Semrock). Images were achieved with NIS-Elements AR software (Nikon) and analyzed by Fiji software [57].

Cell lines were validated by the corresponding repositories. The cell lines were regularly tested for mycoplasma contamination using the MycoProbe Mycoplasma Detection Kit (RnD Systems, Cat#CUL001B), and all the experiments were performed using mycoplasma free cells.

### Quantitative reverse transcriptase–polymerase chain reaction (qRT-PCR)

Total RNA was extracted from two 10-µm-thick liver biopsy sections by using RNeasy FFPE kit (Qiagen), followed by reverse transcription using a MyiQ™ Single-Color Real-Time PCR Detection System (Bio-Rad Laboratories, Hercules, USA). qRT-PCR was performed using SYBR Premix ExTaq (Takara Bio Inc., Kusatsu, Japan). The samples were run in duplicate, and the relative gene expression levels were calculated using the 2^-ΔCt^ method, normalizing to 18SRNA. KiCqStart SYBR Green primers for each gene were purchased from Sigma-Aldrich.

### Western blot

The Q-proteome FFPE Tissue Kit (Qiagen; Cat# 37623) was employed following the manufacturer’s recommendations for protein extraction from fixed liver biopsies. In brief, formalin-fixed paraffin-embedded (FFPE) tissue sections, cut to a thickness of 15 µm, underwent deparaffination in xylene, followed by rehydration through successive washes in 100%, 96%, and 70% ethanol. To remove excess alcohol, the sections were centrifuged at 13,000 rpm for two minutes before the addition of extraction buffers. Then, EXB extraction buffer was added to the tissue pellet and allowed to incubate for 10 minutes on ice. Afterward, the sample was heated to 100 °C for 20 minutes, followed by incubation at 80 °C for 2 hours with shaking at 750 rpm. Finally, the samples was centrifuged at 14,000 xg for 15 minutes at 4 °C, and the supernatant with the extracted proteins was collected.

Detailed methods used for Western blot analysis have been previously described [58]. Rabbit polyclonal anti-caspase-1 p10 (SantaCruz Biotechnology, Dallas, USA; Cat# sc-515) and anti-ASC (SantaCruz Biotechnolgy; Cat# sc-22514-R) were used as primary antibodies, while mouse monoclonal anti-β-actin (SantaCruz Biotechnology; sc-47778) was used as loading control. Immunoblot analysis results were analyzed by densitometry with ImageLab 5.0 software (Bio-Rad Laboratories).

### IL-1β determination

IL-1β was measured by a high sensitivity IL-1β Human ELISA kit (Invitrogen, Carlsbad, USA) following the manufacturer’s instructions and read in a Synergy Mx plate reader (BioTek, Vermont, USA).

### Statistical analysis

Statistics were calculated with GraphPad Prism 8.0.2 software (GraphPad Software Inc., Boston, USA). Data were tested for normal distribution with the Shapiro–Wilk normality test. The homogeneity of data (homoscedasticity) was analyzed with the *F* test. A two-tailed unpaired T test for two-group comparison or ANOVA with the Bonferroni post-test for multiple-group comparison was used wherever parametrical testing applied (normal distribution and homoscedasticity), and the Mann–Whitney test or the Kruskal–Wallis test with the Dunn post-test was used when the dataset had to be analyzed nonparametrically. Correlation analyses were evaluated by using Pearson’s correlation. Survival analysis to assess the outcomes was performed using a survival curve generated based on the Kaplan-Meier method, and the statistical significance of the differences between the survival curves was determined using the log-rank test. No outliers were identified in reported experiments, and no data were excluded from analyses. Given the exploratory nature of this study and the limited availability of participants within the specified timeframe, statistical power calculations were not performed [59]. Two-tailed values of *P* < 0.05 were regarded as significant.

### Supplementary information

Supplementary Figures

Original full-length western blots

## Data Availability

The data that support the findings of this study are available from the corresponding author upon reasonable request. Some data may not be made available because of privacy or ethical restrictions.

## References

[1] Seehofer D, Schoning W, Neuhaus P (2013). Deceased donor liver transplantation. Chirurg.

[2] Asrani SK, Devarbhavi H, Eaton J, Kamath PS (2019). Burden of liver diseases in the world. J. Hepatol.

[3] Saidi RF, Hejazii Kenari SK (2014). Challenges of organ shortage for transplantation: solutions and opportunities. Int J Organ Transpl. Med.

[4] Potter KF, Cocchiola B, Quader MA (2021). Donation after circulatory death: opportunities on the horizon. Curr Opin Anaesthesiol.

[5] Croome KP, Taner CB (2020). The Changing Landscapes in DCD Liver Transplantation. Curr. Transpl. Rep.

[6] Forde JJ, Bhamidimarri KR (2022). Management of Biliary Complications in Liver Transplant Recipients. Clin. Liver Dis.

[7] Rodriguez-Peralvarez M, Rico-Juri JM, Tsochatzis E, Burra P, De la Mata M, Lerut J (2016). Biopsy-proven acute cellular rejection as an efficacy endpoint of randomized trials in liver transplantation: a systematic review and critical appraisal. Transpl. Int.

[8] Luo X, Nicoarä-Farcäu O, Magaz M, Betancourt F, Soy g, Baiges A, et al. Obstruction of the liver circulation. In: Taniguchi T, Lee S, editors. Cardio-Hepatology Connections Between Hepatic and Cardiovascular Disease: Academic Press; 2023. p. 65–92.

[9] Lucas-Ruiz F, Mateo SV, Jover-Aguilar M, Alconchel F, Martinez-Alarcon L, de Torre-Minguela C (2023). Danger signals released during cold ischemia storage activate NLRP3 inflammasome in myeloid cells and influence early allograft function in liver transplantation. EBioMedicine.

[10] Villalba-Lopez F, Garcia-Bernal D, Mateo SV, Vidal-Correoso D, Jover-Aguilar M, Alconchel F (2023). Endothelial cell activation mediated by cold ischemia-released mitochondria is partially inhibited by defibrotide and impacts on early allograft function following liver transplantation. Biomed. Pharmacother.

[11] Barnett KC, Li S, Liang K, Ting JP (2023). A 360 degrees view of the inflammasome: Mechanisms of activation, cell death, and diseases. Cell.

[12] Martinon F, Burns K, Tschopp J (2002). The inflammasome: a molecular platform triggering activation of inflammatory caspases and processing of proIL-beta. Mol. Cell.

[13] de Torre-Minguela C, Mesa Del Castillo P, Pelegrin P (2017). The NLRP3 and Pyrin Inflammasomes: Implications in the Pathophysiology of Autoinflammatory Diseases. Front Immunol.

[14] Lucas-Ruiz F, Penin-Franch A, Pons JA, Ramirez P, Pelegrin P, Cuevas S, et al. Emerging Role of NLRP3 Inflammasome and Pyroptosis in Liver Transplantation. Int J Mol Sci. 2022;23:14396. 10.3390/ijms232214396 PMC9698208 36430874

[15] Wree A, Eguchi A, McGeough MD, Pena CA, Johnson CD, Canbay A (2014). NLRP3 inflammasome activation results in hepatocyte pyroptosis, liver inflammation, and fibrosis in mice. Hepatology.

[16] Gaul S, Leszczynska A, Alegre F, Kaufmann B, Johnson CD, Adams LA (2021). Hepatocyte pyroptosis and release of inflammasome particles induce stellate cell activation and liver fibrosis. J Hepatol..

[17] Stutz A, Horvath GL, Monks BG, Latz E (2013). ASC speck formation as a readout for inflammasome activation. Methods Mol Biol.

[18] Soriano-Teruel PM, Garcia-Lainez G, Marco-Salvador M, Pardo J, Arias M, DeFord C (2021). Identification of an ASC oligomerization inhibitor for the treatment of inflammatory diseases. Cell Death Dis..

[19] Pareja E, Cortes M, Hervas D, Mir J, Valdivieso A, Castell JV (2015). A score model for the continuous grading of early allograft dysfunction severity. Liver Transpl..

[20] Matesanz R, Dominguez-Gil B, Coll E, Mahillo B, Marazuela R (2017). How Spain Reached 40 Deceased Organ Donors per Million Population. Am J Transpl..

[21] Siddiqui F, Al-Adwan Y, Subramanian J, Henry ML (2022). Contemporary considerations in solid organ transplantation utilizing DCD donors. Transplant Rep.

[22] Danion J, Thuillier R, Allain G, Bruneval P, Tomasi J, Pinsard M, et al. Evaluation of Liver Quality after Circulatory Death Versus Brain Death: A Comparative Preclinical Pig Model Study. Int J Mol Sci. 2020;21: 9040. 10.3390/ijms21239040 PMC7730280 33261172

[23] Fu J, Wu H (2023). Structural Mechanisms of NLRP3 Inflammasome Assembly and Activation. Annu Rev. Immunol.

[24] Xiao L, Magupalli VG, Wu H (2023). Cryo-EM structures of the active NLRP3 inflammasome disc. Nature.

[25] Mezzaroma E, Toldo S, Farkas D, Seropian IM, Van Tassell BW, Salloum FN (2011). The inflammasome promotes adverse cardiac remodeling following acute myocardial infarction in the mouse. Proc Natl Acad Sci USA.

[26] Faner R, Sobradillo P, Noguera A, Gomez C, Cruz T, Lopez-Giraldo A, et al. The inflammasome pathway in stable COPD and acute exacerbations. ERJ Open Res. 2016;2:00002–2016. 10.1183/23120541.00002-2016 PMC5034597 27730204

[27] Hong BJ, Liu H, Wang ZH, Zhu YX, Su LY, Zhang MX (2017). Inflammasome activation involved in early inflammation reaction after liver transplantation. Immunol Lett.

[28] Yu Y, Cheng Y, Pan Q, Zhang YJ, Jia DG, Liu YF (2019). Effect of the Selective NLRP3 Inflammasome Inhibitor mcc950 on Transplantation Outcome in a Pig Liver Transplantation Model With Organs From Donors After Circulatory Death Preserved by Hypothermic Machine Perfusion. Transplantation.

[29] Broz P, Dixit VM (2016). Inflammasomes: mechanism of assembly, regulation and signalling. Nat Rev Immunol..

[30] Ruan J, Xia S, Liu X, Lieberman J, Wu H (2018). Cryo-EM structure of the gasdermin A3 membrane pore. Nature.

[31] Kayagaki N, Kornfeld OS, Lee BL, Stowe IB, O’Rourke K, Li Q (2021). NINJ1 mediates plasma membrane rupture during lytic cell death. Nature.

[32] Baroja-Mazo A, Martín-Sánchez F, Gomez AI, Martínez CM, Amores-Iniesta J, Compan V (2014). The NLRP3 inflammasome is released as a particulate danger signal that amplifies the inflammatory response. Nat. Immunol.

[33] Sun P, Zhong J, Liao H, Loughran P, Mulla J, Fu G (2022). Hepatocytes Are Resistant to Cell Death From Canonical and Non-Canonical Inflammasome-Activated Pyroptosis. Cell Mol Gastroenterol Hepatol..

[34] Feng YS, Tan ZX, Wang MM, Xing Y, Dong F, Zhang F (2020). Inhibition of NLRP3 Inflammasome: A Prospective Target for the Treatment of Ischemic Stroke. Front Cell Neurosci.

[35] Han PP, Han Y, Shen XY, Gao ZK, Bi X (2023). NLRP3 inflammasome activation after ischemic stroke. Behav Brain Res.

[36] Zheng Y, Xu L, Dong N, Li F (2022). NLRP3 inflammasome: The rising star in cardiovascular diseases. Front Cardiovasc Med.

[37] Wang Y, Huang H, He W, Zhang S, Liu M, Wu S (2021). Association between serum NLRP3 and malignant brain edema in patients with acute ischemic stroke. BMC Neurol.

[38] Cheng X, Zhao H, Wen X, Li G, Guo S, Zhang D (2023). NLRP3-inflammasome inhibition by MCC950 attenuates cardiac and pulmonary artery remodelling in heart failure with preserved ejection fraction. Life Sci..

[39] Toldo S, Mezzaroma E, Buckley LF, Potere N, Di Nisio M, Biondi-Zoccai G (2022). Targeting the NLRP3 inflammasome in cardiovascular diseases. Pharm. Ther.

[40] Pollara J, Edwards RW, Lin L, Bendersky VA, Brennan TV (2018). Circulating mitochondria in deceased organ donors are associated with immune activation and early allograft dysfunction. JCI Insight.

[41] Qiao J, Wu X, Luo Q, Wei G, Xu M, Wu Y (2018). NLRP3 regulates platelet integrin alphaIIbbeta3 outside-in signaling, hemostasis and arterial thrombosis. Haematologica.

[42] Martin-Sanchez F, Compan V, Penin-Franch A, Tapia-Abellan A, Gomez AI, Banos-Gregori MC (2023). ASC oligomer favors caspase-1CARD domain recruitment after intracellular potassium efflux. J Cell Biol.

[43] Tapia-Abellan A, Angosto-Bazarra D, Alarcon-Vila C, Banos MC, Hafner-Bratkovic I, Oliva B (2021). Sensing low intracellular potassium by NLRP3 results in a stable open structure that promotes inflammasome activation. Sci Adv.

[44] Jimenez-Castro MB, Cornide-Petronio ME, Gracia-Sancho J, Peralta C (2019). Inflammasome-Mediated Inflammation in Liver Ischemia-Reperfusion Injury. Cells.

[45] van Beekum CJ, Vilz TO, Glowka TR, von Websky MW, Kalff JC, Manekeller S (2021). Normothermic Machine Perfusion (NMP) of the Liver - Current Status and Future Perspectives. Ann. Transpl..

[46] Dengu F, Abbas SH, Ebeling G, Nasralla D. Normothermic Machine Perfusion (NMP) of the Liver as a Platform for Therapeutic Interventions during Ex-Vivo Liver Preservation: A Review. J Clin Med. 2020;9. 10.3390/jcm9041046 PMC7231144 32272760

[47] Matos LL, Trufelli DC, de Matos MG, da Silva Pinhal MA (2010). Immunohistochemistry as an important tool in biomarkers detection and clinical practice. Biomark. Insights.

[48] O’Hurley G, Sjostedt E, Rahman A, Li B, Kampf C, Ponten F (2014). Garbage in, garbage out: a critical evaluation of strategies used for validation of immunohistochemical biomarkers. Mol. Oncol..

[49] Dunstan RW, Wharton KA, Quigley C, Lowe A (2011). The use of immunohistochemistry for biomarker assessment-can it compete with other technologies?. Toxicol. Pathol..

[50] Nistor A, Watson PH, Pettigrew N, Tabiti K, Dawson A, Myal Y (2006). Real-time PCR complements immunohistochemistry in the determination of HER-2/neu status in breast cancer. BMC Clin. Pathol.

[51] Hessheimer AJ, Coll E, Torres F, Ruiz P, Gastaca M, Rivas JI (2019). Normothermic regional perfusion vs. super-rapid recovery in controlled donation after circulatory death liver transplantation. J. Hepatol..

[52] Watson CJE, Hunt F, Messer S, Currie I, Large S, Sutherland A (2019). In situ normothermic perfusion of livers in controlled circulatory death donation may prevent ischemic cholangiopathy and improve graft survival. Am. J. Transpl.

[53] Hessheimer AJ, de la Rosa G, Gastaca M, Ruiz P, Otero A, Gomez M, et al. Abdominal normothermic regional perfusion in controlled donation after circulatory determination of death liver transplantation: Outcomes and risk factors for graft loss. Am J Transplant. 2021. 10.1111/ajt.16899 34856070

[54] Cascales-Campos PA, Ferreras D, Alconchel F, Febrero B, Royo-Villanova M, Martinez M (2020). Controlled donation after circulatory death up to 80 years for liver transplantation: Pushing the limit again. Am J Transpl.

[55] Ormonde DG, de Boer WB, Kierath A, Bell R, Shilkin KB, House AK (1999). Banff schema for grading liver allograft rejection: utility in clinical practice. Liver Transpl Surg..

[56] Barbera-Cremades M, Baroja-Mazo A, Gomez AI, Machado F, Di Virgilio F, Pelegrin P (2012). P2X7 receptor-stimulation causes fever via PGE2 and IL-1beta release. FASEB J.

[57] Schindelin J, Arganda-Carreras I, Frise E, Kaynig V, Longair M, Pietzsch T (2012). Fiji: an open-source platform for biological-image analysis. Nat Methods.

[58] Baroja-Mazo A, Compan V, Martin-Sanchez F, Tapia-Abellan A, Couillin I, Pelegrin P (2019). Early endosome autoantigen 1 regulates IL-1beta release upon caspase-1 activation independently of gasdermin D membrane permeabilization. Sci Rep.

[59] Kunselman AR. A brief overview of pilot studies and their sample size justification. Fertil Steril. 2024. 10.1016/j.fertnstert.2024.01.040 PMC11128343 38331310

