# Supplementary material for: Detection of inflammasome activation in liver tissue during the donation process as potential biomarker for liver transplantation
Source: Cell Death Discov. 2024 May 30;10:266. doi: 10.1038/s41420-024-02042-y (PMC11139956; doi:10.1038/s41420-024-02042-y)
Supplement: Supplementary file 1 — Supplementary Figures [file 41420_2024_2042_MOESM1_ESM.pdf]

Supplementary Figure 1

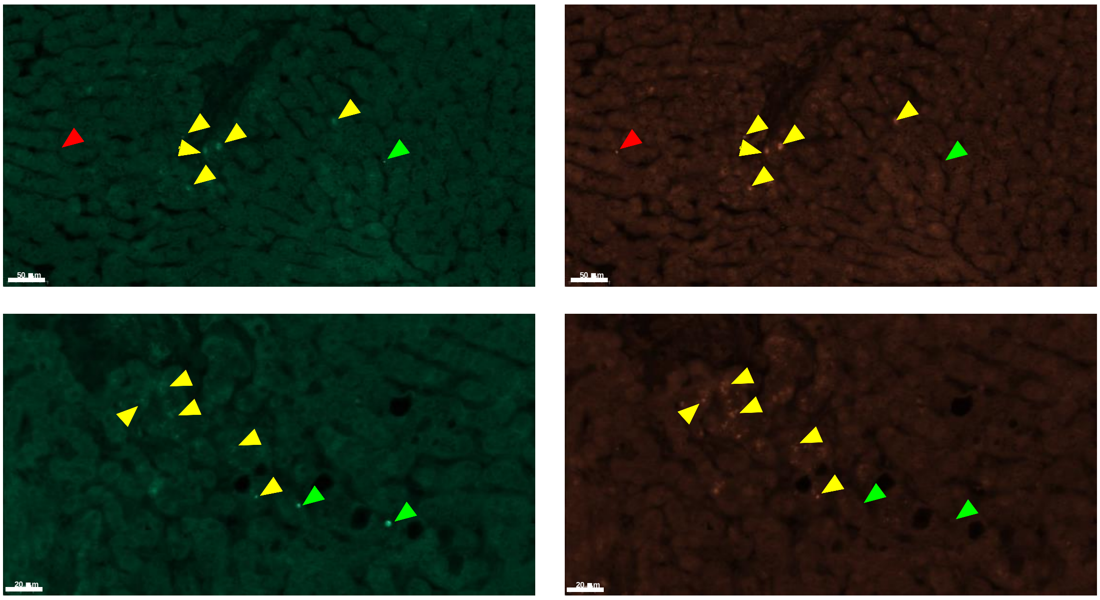

Figure S1.- Single staining of liver biopsies for ASC and cleaved IL-1β. Liver biopsy samples were double counter-stained with anti-ASC (green) and anti-active IL-1β (red) (Invitrogen; #PA5-105048). Green arrowheads signal single staining for ASC specks, red arrowheads indicate the presence of single staining for active IL-1β, while yellow arrowheads show co-staining for both markers (see Figure 2d).

Supplementary Figure 2

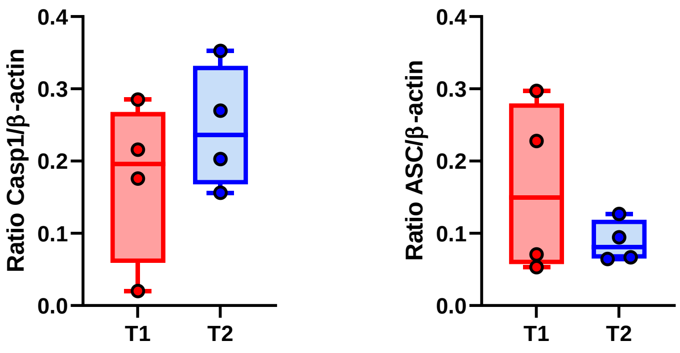

Figure S2.- Detection of ASC and caspase-1 in liver biopsies by Western Blot. Ratio between detected caspase-1 and ASC with the loading control protein β-actin as calculated by densitometry using the ImageLab 5.0 software (Bio-Rad).
