# Supplementary figures and images for: Detection of inflammasome activation in liver tissue during the donation process as potential biomarker for liver transplantation
Source: Cell Death Discov. 2024 May 30;10:266. doi: 10.1038/s41420-024-02042-y (PMC11139956; doi:10.1038/s41420-024-02042-y)

## Original full-length Western blots

Figure 6 A

Caspase 1

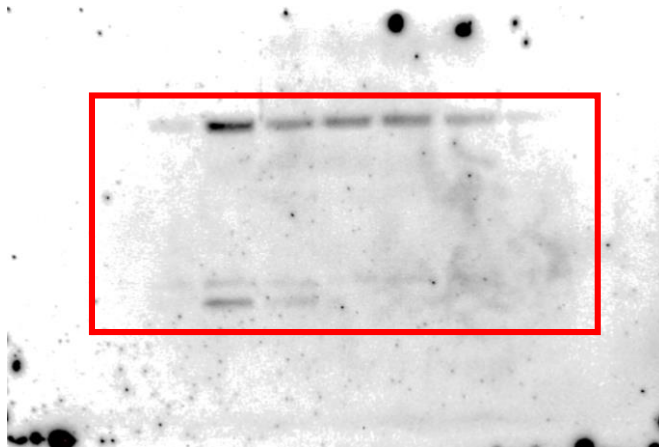

ASC

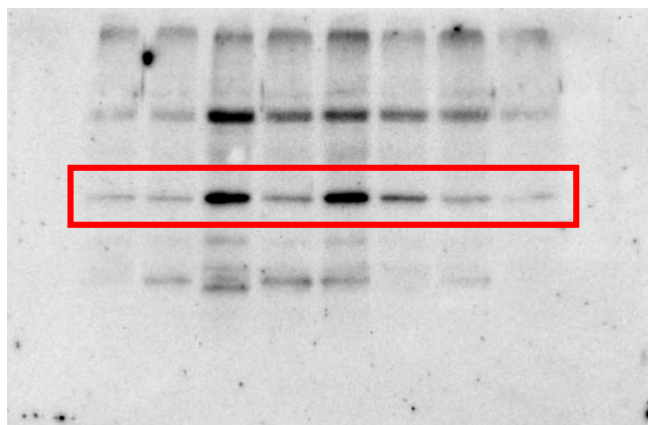

$\beta$ -actin

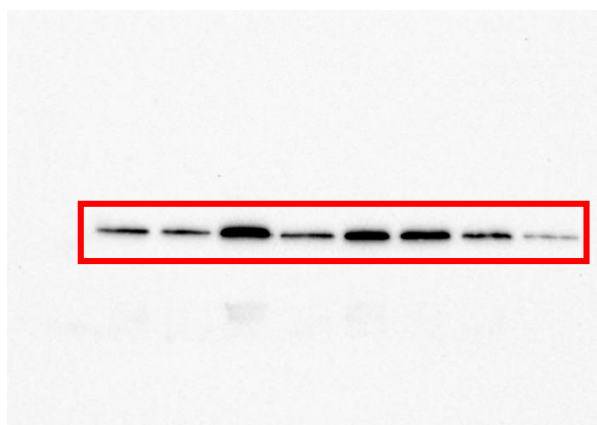

Supplement: Supplementary file 2 — Original full-length western blots [file 41420_2024_2042_MOESM2_ESM.pdf]
